# Optimal VLBI baseline geometry for UT1-UTC Intensive observations

**DOI:** 10.1007/s00190-021-01530-8

**Published:** 2021-06-19

**Authors:** Matthias Schartner, Lisa Kern, Axel Nothnagel, Johannes Böhm, Benedikt Soja

**Affiliations:** 1 ETH Zürich, Robert-Gnehm-Weg 15, CH-8093 Zürich, Switzerland; 2 TU Wien, Wiedner Hauptstrasse 8, A-1040 Wien, Austria

**Keywords:** VLBI, dUT1, Intensive sessions

## Abstract

One of the main tasks of Very Long Baseline Interferometry (VLBI) is the rapid determination of the highly variable Earth’s rotation expressed through the difference between Universal Time UT1 and Coordinated Universal Time UTC (dUT1). For this reason, dedicated one hour, single baseline sessions, called *“Intensives”*, are observed on a daily basis. Thus far, the optimal geometry of *Intensive* sessions was understood to include a long east–west extension of the baseline to ensure a dUT1 estimation with highest accuracy. In this publication, we prove that long east–west baselines are the best choice only for certain lengths and orientations. In this respect, optimal orientations may even require significant inclination of the baseline with respect to the equatorial plane. The basis of these findings is a simulation study with subsequent investigations in the partial derivatives of the observed group delays $\tau$ with respect to dUT1 $\partial \tau /\partial dUT1$. Almost 3000 baselines between artificial stations located on a regular $10 \times 10$ degree grid are investigated to derive a global and generally valid picture about the best length and orientation of *Intensive* baselines. Our results reveal that especially equatorial baselines or baselines with a center close to the equatorial plane are not suited for *Intensives* although they provide a good east–west extension. This is explained by the narrow right ascension band of visible sources and the resulting lack of variety in the partial derivatives. Moreover, it is shown that north–south baselines are also capable of determining dUT1 with reasonable accuracy, given that the baseline orientation is significantly different from the Earth rotation axis. However, great care must be taken to provide accurate polar motion a priori information for these baselines. Finally, we provide a better metric to assess the suitability of *Intensive* baselines based on the effective spread of $\partial \tau /\partial dUT1$.

## Introduction

Very Long Baseline Interferometry (VLBI) is critical for the realization of the International Terrestrial Reference Frame (ITRF) (Altamimi et al. 2016) and the International Celestial Reference Frame (ICRF) (Charlot et al. 2020), and it is the only space geodetic technique capable of consistently estimating all Earth orientation parameters (EOPs) (Petit and Luzum 2010) representing the transformation parameters between the ITRF and the ICRF. In particular, the determination of the highly variable Earth’s phase of rotation, parametrized as difference between Universal Time (UT1) and Coordinated Universal Time (UTC) ($dUT1=UT1-UTC$), is ensuring the unique position of VLBI. This aspect leads to the importance of so-called *Intensives* on a daily basis, which are one-hour VLBI sessions that have been carried out since 1984 (Robertson et al. 1985).

Thus far, it has been common knowledge that long east–west baselines provide the best sensitivity for this product. The concept of the long east–west baselines stems from the fact that for these baselines and an observation in the equatorial plane normal to the baseline ($\alpha = 90^\circ$), the impact of a small rotation angle $d\beta$ on the observed delay $\tau$ scales with the baseline length *b*:

1 $$\begin{aligned} d\tau = b \cdot \sin \alpha \, d\beta . \end{aligned}$$

For a baseline of length equal to the Earth radius (${6370}\hbox { km}$) and an observation perpendicular to the baseline, an error in the delay observable $\tau$ of 33 picoseconds (${33}\hbox { ps} \equiv 1 \hbox { cm}$) corresponds to an angular misorientation of the baseline of 21.6 microseconds (${\mu } \hbox {s}$), which may be considered equivalent to an error in dUT1. If the baseline length is doubled ($2R = 12\,740 \hbox { km}$), the same delay error of ${33}\hbox { ps}$ produces an error of ${10.8}\,{\mu } \hbox {s}$ in dUT1. Consequently, with longer baselines we can better resolve changes in the rotation angle.

As we will explain below, this is only half of the story because for producing dUT1 results, we need to estimate more parameters, namely parameters describing tropospheric zenith delays above and clock differences between the stations. For this purpose, redundant observations are performed within one hour and a least squares adjustment is computed. Additionally, it is impossible to collect observations on very long baselines close to $12\, 740 \hbox { km}$ lengths, since there will be no mutually visible radio sources and thus the delay error will increase again with very long baseline lengths. This is where the concept has to be seen in a slightly different way.

Over the last decade, various research studies have been carried out to improve VLBI *Intensives*. Here, we should recall that the *“Intensive”* sessions were initiated to keep the requirements for the logistics much smaller and the turn around times for product delivery much shorter than for 24-hour network sessions. This resulted in the single baseline and one hour only observing setup which inadvertently led to a reduced precision. Thus, the network geometry and scheduling process of *Intensives* are crucial. To overcome the problem of the lack of precision concerning *Intensives* in comparison with regular 24h VLBI sessions, several approaches were discussed in the past.

Artz et al. (2012) showed that doubling the session duration to two hours improves the dUT1 formal error by a factor of $\sqrt{2}$ at the cost of increased data collection and thus latency. Increasing the number of observations and thus improving the dUT1 formal error by including additional telescopes was also investigated in several studies. Leek et al. (2015) assessed the benefits of using twin telescopes as well as developing a new scheduling approach based on impact factors to improve the observing geometry. A different approach was suggested by Kareinen et al. (2017), who analyzed the impact of adding a tag-along station to *“Intensive”* sessions. Simulated observations with a three-station network resulted in an improvement of up to 40% in dUT1 in the yearly weighted root mean square error compared to a two-station network. However, by adding more telescopes the amount of recorded data increases, likely resulting in an increased data transfer and processing duration and thus degraded dUT1 latency.

There have been suggestions for scheduling-based approaches to improve *“Intensive”* sessions. Uunila et al. (2012) examined the effect of the source constellation on the quality of the estimation of UT1. They made use of the concept of relating all observations to a baseline mid-point as proposed by Nothnagel and Campbell (1991) and showed that observations located at the corners of the mutually visible sky of the stations are crucial for achieving the highest dUT1 sensitivity. Later, Baver and Gipson (2015) implemented a minimization algorithm based on the observation gradient to minimize the dUT1 formal error while also investigating the differences in using all sources versus a small set of strong sources. In that study, it was further confirmed that observations at the corners of the mutually visible sky are important for the geometric stability of the estimation of dUT1 and therefore help to minimize the formal error.

Various investigations were devoted to a proper source selection for *Intensives*. Baver and Gipson (2014) investigated the impact of different source lists and highlighted the effect on dUT1 formal errors as well as on the vulnerability to atmospheric turbulence and source loss. Later, an alternative approach of using all mutually visible sources was developed, called the “Maximum Source Strategy”. Over two years, *Intensives* were observed alternating between the standard and the new source list. Gipson and Baver (2016) compared the results and concluded—based on this data set and based on dedicated R&D experiments—that the new approach performed as well as or even better than the original strategy, depending on the metric applied. More recently, Baver and Gipson (2020) showed that balancing source strength and sky coverage in a newly defined set of sources (called “Balanced 50”) leads to an improvement of the weighted dUT1 formal error by ${2.6}\,{\mu } \hbox {s}$ in comparison with using all visible sources in the process of scheduling.

Most recently, Corbin et al. (2020) presented a mixed-integer linear programming method to schedule VLBI *Intensives* by selecting a schedule that maximizes the sky coverage among all possible schedules. For this purpose, a new sky coverage score is introduced, based on a hierarchical partitioning of the sky above the telescopes into cells of equal surface area. This method increases the number of observations and improves the simulated precision of dUT1 in comparison with standard schedules at the cost of a significantly increased computation time.

While most of the studies listed above tried to improve existing VLBI *Intensives* based on improved scheduling, a better source selection, or by increasing the number of observations through adding more telescopes or longer session duration, this study aims at identifying the best geometry of a two-station VLBI *“Intensive”* baseline. Following the approach used by Schartner et al. (2020) of evaluating artificial antennas to investigate impacts of network geometry on a global scale, artificial antennas are placed on a regular $10 \times 10$ degree grid and all baselines between these artificial antennas are individually investigated (see Sect. 2.3). Section 3 elaborates on the simulation results of almost 3000 baselines, while Sect. 4 interprets the results based on this investigation, especially by assessing the partial derivatives of $\tau$ with respect to dUT1 $\left( \frac{\partial \tau }{\partial dUT1} \right)$. Finally, Sect. 5 summarizes the findings.

## Method

### Least-squares approach

Within this work, VLBI *Intensives* are simulated to investigate their sensitivity with respect to dUT1. For the analysis, a standard least squares adjustment is considered. In this case, $n_{obs}$ linearized observation equations

2 $$\begin{aligned} \mathbf {v} = A \, \mathbf {dx} - \mathbf {l} \end{aligned}$$

need to be solved, with $\mathbf {v}$ being the residual vector, *A* the Jacobian matrix, $\mathbf {dx}$ the corrections for the unknowns $\mathbf {x}$ concerning the a priori values $\mathbf {x_0}$ ($\mathbf {x} = \mathbf {x_0}+\mathbf {dx}$) and $\mathbf {l}$ denoting the vector of “observed” minus “computed values”. The goal of the least squares method is to find a solution of $\mathbf {dx}$ that minimizes the weighted square sum of the residuals $\mathbf {v}$

3 $$\begin{aligned} \min {\left( \mathbf {v}^\top P \mathbf {v} \right) } \end{aligned}$$

with *P* denoting the weight matrix for the observations. This leads to the so-called normal Eq. 4 with the normal equation matrix *N* (Eq. 5) and the right-hand side $\mathbf {b}$ (Eq. 6).

4 $$\begin{aligned} N \mathbf {dx}&= \mathbf {b} \end{aligned}$$

5 $$\begin{aligned} N&= A^\top P A \end{aligned}$$

6 $$\begin{aligned} \mathbf {b}&= A^\top P \mathbf {l} \end{aligned}$$

The variance-covariance matrix $\varSigma$ of the unknown parameters is the inverse of the normal equation matrix

7 $$\begin{aligned} \varSigma _{xx} = N^{-1} \end{aligned}$$

and the standard deviation $\sigma$ of an unknown parameter is found as the square root of the corresponding element in the main diagonal of $\varSigma _{xx}$. Typically, the variance-covariance matrix $\varSigma$ is multiplied by the a posteriori variance factor $s_0^2$. However, in this work, we only want to examine the optimal baseline geometry excluding the effects of any conflicting variations in the variances of the observations. For this reason, we decided to ignore this factor by setting it to one. Future investigations will deal with sophisticated Monte Carlo simulations (Pany et al. 2011) where the a posteriori variance factor will play a major role as well.

### *Intensive* scheduling approach

Within this work, we aim at identifying the geometry of the best possible baseline for the determination of dUT1. For this purpose, great care was taken to produce highly optimized schedules to ensure a fair comparison of all potential baselines. Using VieSched++ (Schartner and Böhm 2019), monthly 1h-long schedules, starting at 07:00 UTC on the first day of each month, were generated using a source list of approximately 300 sources which are especially well suited for geodetic VLBI. Since baselines between VGOS-type telescopes are investigated (see Sect. 2.3), the observation duration was fixed to ${30}\hbox { s}$, while the slew time was calculated rigorously to mimic the current VGOS observation strategy.

Following the suggestions of Uunila et al. (2012) and Baver and Gipson (2015), the best approach to schedule VLBI *Intensives* is to focus on observations of radio sources located at the corners of the mutually visible sky. For this particular reason, a special scheduling algorithm implemented in VieSched++ was used. The general idea of the scheduling algorithm is that it starts by observing a source located at the corner of the mutually visible sky, and after a certain number of seconds $\delta t_c$ (that can be defined by the user), the algorithm drastically increases the likelihood of observing a source located at the opposite corner of the mutually visible sky. In between these scans, the scheduler is free to pick any source following the standard geodetic scheduling rules (see Schartner and Böhm (2019) for more information about the standard geodetic scheduling rules).

This study investigates *Intensive* sessions with fast-slewing VGOS telescopes (see Sect. 2.3) where a value of $\delta t_c$ between ${600}\hbox { s}$ and ${900}\hbox { s}$ is most beneficial. In this study, we were using a value of ${900} \hbox { s}$, while other Intensive sessions between Ishioka (Japan) and Onsala (Sweden) were already observed with $\delta t_c={600}\hbox { s}$ (Haas et al. 2021). For slower slewing legacy SX telescopes that are mostly used for the IVS *Intensives*, a larger value of $\delta t_c$ is preferable. However, this highly depends on the telescope properties and needs to be tested individually.

A detailed description of the scheduling strategy can be found in Appendix A.

### Experiment setup

To identify the optimal baseline geometry for *Intensives*, artificial antennas were placed on a regular $10 \times 10$ degrees latitude (*lat*)–longitude (*lon*) grid. The antennas were assumed to have the same properties (e.g., slew speeds) as the WETTZ13S telescope; thus, they are VGOS-type telescopes (Petrachenko et al. 2012; Niell et al. 2018). The latitude limits of the artificial telescope grid were set to $-80^\circ$ and $+80^\circ$ resulting in a total of 17 different latitude levels.

**Fig. 1:**
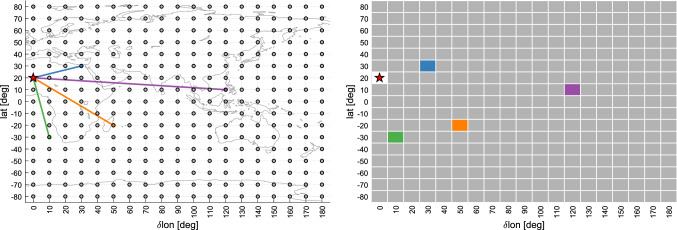
Sketch of the experiment setup. One station is held as the reference station (highlighted by a red star). Every baseline between the reference station and any other station (gray circle) is investigated. The performance of a baseline is visualized in the grid cell that corresponds to the location of the second station. Four randomly selected baselines are highlighted for illustration purposes

Since the Earth is (to a first approximation) rotationally symmetric, it is permissible to only investigate baselines with one station located at an arbitrary reference meridian (e.g., zero longitude) and it is sufficient to only investigate a total $\varDelta$-longitude baseline range of 180 degrees to derive a universal and global conclusion, assuming that multiple sessions, distributed over a full year with a fixed session start time, are investigated. The reason for these valid simplifications can be explained by the following considerations: Any baseline on Earth can be rotated around the z-axis so that it has the first telescope on the zero meridian and the second one in the East of that. By cycling the observing schedules with fixed start times over a full year, they comprise all possible changes in sidereal time having an effect on possible source selections. Therefore, a mean baseline sensitivity in dUT1 can be expressed based on investigating a full year of sessions with a fixed start time.

Thus, the longitude limits of the artificial telescope grid limits were set to $0^\circ$ and $180^\circ$ with $0^\circ$ being the reference longitude resulting in a total of 19 different longitude levels and a total of $17 \cdot 19 = 323$ artificial remote telescopes. On the reference meridian, the nine stations with $lat \ge 0$ were selected as the reference stations. Reference stations with $lat < 0$ were not investigated since their performance is approximately identical to the same baseline mirrored at the equator. The only difference that might occur is due to the difference of available good radio sources in the southern hemisphere compared to the northern hemisphere, which is known to be less dense while having higher uncertainties (Plank et al. 2015; Charlot et al. 2020). However, the difference is neglected in this study.

From the reference stations, every baseline to any artificial remote station was investigated. This leads to 322 possible baselines per reference station. Since nine reference stations need to be considered, the total number of baselines is $9 \cdot 322 = 2898$. For every baseline, one schedule per month was generated, resulting in a total of ${34\,776}$ schedules to be analyzed. Figure 1 illustrates the situation for the reference station at a latitude of 20 degrees.

**Fig. 2:**
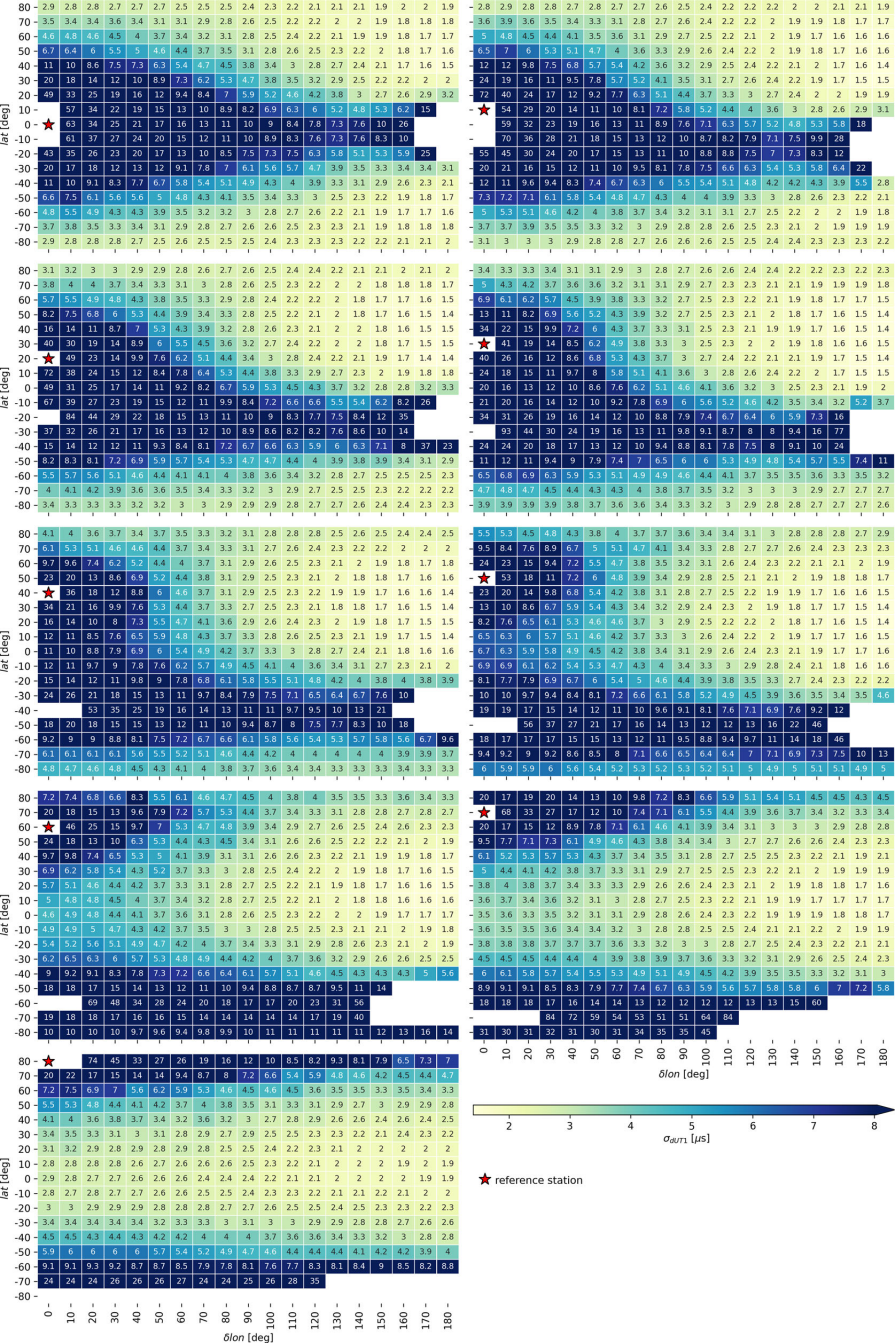
Performance of every investigated baseline in terms of standard deviation $\sigma _{dUT1}$. In each sub-graph, the reference station is highlighted by a red star. White areas mark baselines that did not provide sensible results in the analysis. The average dUT1 precision on the corresponding baseline is color-coded (see Fig. 1) and added as number to the cells

The gray dots represent the artificial stations, the red star marks the reference station itself. For comparison, the coastlines are displayed in the background. However, one has to keep in mind that the station grid can be rotated around the z-axis (and thus shifting the longitudes) at will without changing the interpretation of the results as discussed previously. The right plot in Fig. 1 highlights how the results, in particular in Fig. 2, need to be interpreted. For this purpose, four randomly selected baselines are displayed and their corresponding grid cells are color-coded.

## Results

The results of all simulations consist of a wealth of variance-covariance matrices $\varSigma _{xx}$ for each of the twelve schedules per baseline equally distributed over one year calculated according to Sect. 2.1. From $\varSigma _{xx}$ the standard deviation of dUT1 $\sigma _{dUT1}$ was extracted applying an a posteriori variance factor of one as mentioned above. The mean $\sigma _{dUT1}$ over the twelve different schedule start times was taken as the metric to compare the individual baselines.

As expected, there are variations in terms of $\sigma _{dUT1}$ between the twelve different schedules. For most baselines ($80\%$), the standard deviation of the estimated $\sigma _{dUT1}$ values from the twelve schedules is below $20\%$ of the mean value. For $25\%$ of all baselines, it is below $10\%$. Very short and very long baselines show the most variation since either the scheduling or the analysis fails in some cases.

The results of the mean $\sigma _{dUT1}$ over the twelve different schedules can best be interpreted using graphical displays (Fig. 2). Every grid cell contains the mean standard deviation $\sigma _{dUT1}$ and represents the baseline between the reference station (red star) and the remote station located at the position of the grid cells. White areas mark baselines that did not provide estimates in the analysis.

There are two reasons why a baseline might fail to provide a $\sigma _{dUT1}$ value. The first reason is a lack of valid observations and the resulting singularity of *N* in the analysis. This is the case for very long baselines with lengths close to the Earth’s diameter. In this case, the two stations are on the opposite side of the Earth and there is no commonly visible sky, especially when considering that the minimum elevation angle for valid observations was set to five degrees. Examples for this situation are baselines between $(lat,\, \delta lon)=(0,\, 0)$–$(0,\, 180)$ or $(40,\, 0)$–$(-40,\, 180)$.

The second reason is that baselines that are oriented (close to) parallel to the Earth rotation vector (which is approximately the z-axis). Examples for these baselines are $(20,\, 0)$–$(-20,\, 0)$ or $(60,\, 0)$–$(-60,\, 0)$. In this case, the baseline is not sensitive to dUT1 and thus fails to estimate it during the analysis, compare Sect. 4 for further discussion on this topic.

The results of Fig. 2 deserve some detailed discussions. In general, there are three groups of baselines leading to high dUT1 formal errors. The first group contains short baselines. As expected, they are not sensitive enough to provide good accuracy. The second group is made up by very long baselines. As discussed earlier, this can be explained by the limitation of the mutually visible sky and the resulting lack of commonly visible sources. Investigations of the number of observations per baselines revealed that due to the fast slew times of the VGOS-style telescopes, almost all schedules have an equal number of observations except for very long baselines where the number of observations drastically decreased explaining the degradation of $\sigma _{dUT1}$. The third group comprises baselines whose mid-point is close to the equatorial plane. If the following condition is met

8 $$\begin{aligned} lat_1 + lat_2 \approx 0 \end{aligned}$$

the resulting dUT1 precision decreases drastically. Consequently, a baseline located exactly at the equator will provide very bad dUT1 formal errors even though it has a perfect east–west orientation. The reason for this behavior is a lack of variability in $\frac{\partial \tau }{\partial dUT1}$ explained by the narrow right ascension band of visible sources. This topic will be further discussed in Sect. 4.

Optimal baselines are defined by the minimum $\sigma _{dUT1}$ in each sub-graph. There are many baseline candidates that are providing reasonably good simulated dUT1 mean formal errors of ${<2}\,{\mu } \hbox {s}$. The global minimum of $\sigma _{dUT1}$ can be identified as ${1.4}\,{\mu } \hbox {s}$. Multiple baselines, including reference station latitudes between 10 and 50 degrees, manage to achieve this accuracy level. In these cases, the optimal counterpart location is identified at $\delta lon = 180$ degrees and at mid-latitudes. As a rule of thumb, one can conclude that it is beneficial to have one station in a high-to-mid-latitude range between $40^\circ$ and $60^\circ$ and the second station at a low-to-mid-latitude range between $10^\circ$ and $40^\circ$ while having a large $\delta lon$.

Surprisingly at first sight, in case the baseline has a pure north–south orientation, while one station is located close to the pole, e.g., between $(lat,\, \delta lon)=(80,\, 0)$–$(0,\, 0)$, the resulting precision is only 50 percent worse compared to the most perfect baseline with a high latitude station that is $(80,\, 0)$–$(0,\, 180)$ and far better than pure east–west baselines located at the equator or any baselines centered around the equator. Thus, it is not strictly necessary to have a long east–west extension to derive reasonable dUT1 accuracy. However, as will be discussed in Sect. 3.1, highly accurate polar motion a priori information is necessary for estimating dUT1 from north–south baselines.

As a reference, Table 1 lists the most commonly observed baselines of the IVS *Intensive* programs from the years 2019–2021 as listed in the *Intensive* schedule master^1^

**Table 1:** List of the most common IVS *Intensive* baselines and their corresponding theoretical dUT1 mean formal errors based on the baseline geometry assuming VGOS-style telescopes. The column “count” refers to the number of times this baseline is listed in the *Intensive* schedule master of the years 2019–2021. The column $lat_{ref}$ contains the reference latitudes (red star in Fig. 2) and *lat* the latitudes of the second station. The parameters $lat_{ref}$, *lat* and $\delta lon$ are in degrees, while $\sigma _{dUT1}$ is in ${\mu } \hbox {s}$. The latitudes of the stations forming the baseline marked with an asterisk (*) are mirrored at the equator as discussed in Sect. 2.3

baseline	count	$lat_{ref}$	*lat*	$\delta lon$	$\sigma _{dUT1}$
KK-WZ	899	20	50	170	1.5
WZ-IS	367	50	40	130	1.9
MK-WZ	251	20	50	170	1.5
NY-WZ	203	80	50	0	5.5
PT-WZ	144	30	50	120	2.1
MK-PT	111	20	30	50	6.0
WZ-SH	107	50	30	110	2.3
NY-IS	103	80	40	130	2.6
WZ-SV	62	50	60	20	15.0
OE-IS	57	60	40	130	2.2
SV-KK	56	60	20	170	1.6
NY-SH	44	80	30	110	2.5
KK-NY	20	20	80	170	2.1
SH-IS	19	30	40	20	15.0
AG-WZ*	16	30	-50	70	7.0
KK-WF	16	20	40	90	2.8
WF-WZ	15	40	50	80	3.1
KK-GS	12	20	40	80	3.2
GS-WZ	10	40	50	90	2.9

To evaluate the geometry of the baselines, their corresponding simulated dUT1 standard deviations from Fig. 2 are listed, along with the position of these baselines within Fig. 2. Thus, station properties of the real telescopes are ignored in this comparison since the proposed standard deviations are still based on assuming artificial VGOS-style telescopes. Column $lat_{ref}$ can be used to identify the subplot within Fig. 2, while column *lat* and $\delta lon$ can be used to find the position of the baseline within this subplot.

**Fig. 3:**
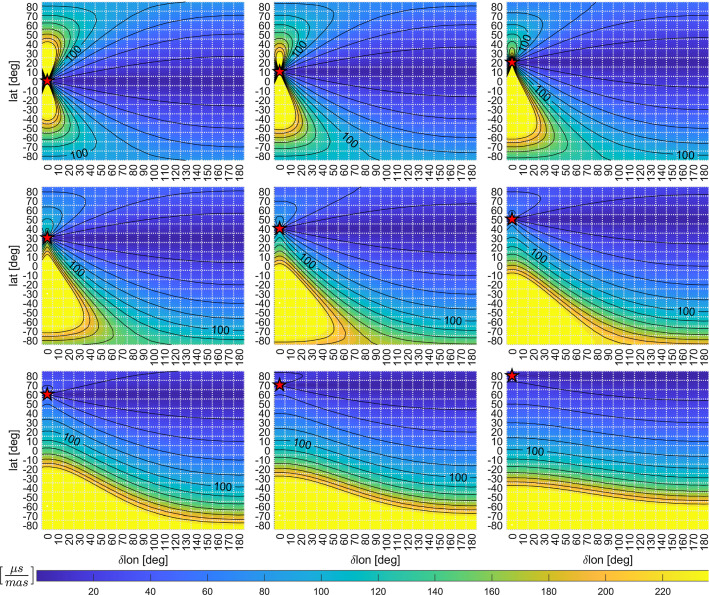
Impact of erroneous PM a priori information (combined impact by x- and y-pole) on dUT1 in units of $\mu s/mas$ averaged over 36 different reference longitudes $\{0,\;10,\;20,\;\dots \;350\}$. In each sub-graph, the reference station is highlighted by a red star

The stations are: KK (Kokee, USA), WZ (Wettzell, Germany), IS (Ishioka, Japan), MK (Mauna Kea, USA), NY (Ny-Ålesund, Norway), PT (Pie Town, USA), SH (Seshan, China), SV (Svetloe, Russia), OE (Onsala, Sweden), AG (Aggo, Argentina), WF (Westford, USA), GS (GGAO, USA). In case of stations with multiple telescopes, only one representative telescope is selected (e.g., for KK, WZ, and OE).

It can be seen that the frequently scheduled baselines KK-WZ and MK-WZ are among the best ones available with a theoretical mean formal error of ${1.5}\,{\mu } \hbox {s}$. Other commonly scheduled baselines, such as WZ-IS and PT-WZ, are also at suitable locations with mean formal errors of ${1.9}\,{\mu } \hbox {s}$ and ${2.1}\,{\mu } \hbox {s}$, respectively. The baseline NY-WZ is also commonly listed in the *Intensives* schedule master. The reason for this is that this baseline is mostly part of the INT3 sessions, where up to five stations are participating—some of which are forming well performing baselines such as WZ-IS and WZ-SH. Table 1 shows that the mean formal error for the baseline NY-WZ is worse compared to other baselines, but it is not as bad as one would have expected for that type of baseline.

However, it is to note that this comparison is only based on the geometry of the baseline and does not reflect the real performance of the corresponding *Intensive* sessions properly. For this, it would also be necessary to consider other factors beyond the scope of this study such as the telescope sensitivities, slew speeds, recording rates and horizon masks.

### Intermezzo: impact of erroneous polar motion a priori information

*Intensive* sessions are typically single baselines sessions. Thus, the number of observations is relatively small and only a subset of parameters can be estimated during the analysis. In general, polar motion (PM) is not estimated and thus has to be fixed to a pair of a priori values. Errors in the PM a priori values will propagate to the estimated dUT1 values, impacting their accuracy. Following Nothnagel and Schnell (2008), the impact of PM errors depends on the baseline geometry and can be assessed based on the derivatives

9 $$\begin{aligned} \frac{dUT1}{dx_p}&= - \frac{(y_2-y_1)(z_2-z_1)}{(y_2-y_1)^2+(x_2-x_1)^2} \qquad [-] \end{aligned}$$

10 $$\begin{aligned} \frac{dUT1}{dy_p}&= - \frac{(x_2-x_1)(z_2-z_1)}{(y_2-y_1)^2+(x_2-x_1)^2} \qquad [-] \end{aligned}$$

where $dx_p$ and $dy_p$ are the uncertainties in the PM a priori values and *x*, *y*, and *z* are the coordinates of the two stations forming the baseline.

The combined impact of Eqs. 9 and 10 in units of $\mu s/mas$ is visualized in Fig. 3. The position of the reference station is highlighted by a red star. The blue areas depict the regions for the second telescope where the impact is minimal.

In contrast to the investigation of the best baseline geometry, where only the longitudinal difference is of importance, Eqs. 9 and 10 directly depend on the station longitudes. To gain information about an average impact, Fig. 3 depicts the average of the estimates of 36 different reference longitudes $\{0,\;10,\;20,\;\dots \;350\}$.

Looking at Fig. 3 and Eqs. 9 and 10, it is evident that especially dUT1 estimates from baselines with a big difference in the z-coordinate between the stations are most affected by erroneous PM a priori information. Thus, great care must be taken when observing dUT1 from these baselines to ensure highly accurate a priori information. Combined estimation of dUT1 and PM from VLBI Intensives and a global navigation satellite system (GNSS) network might be advisable for this task (Hellmers et al. 2019).

The most heavily affected baselines also perform poorly based on the evaluation of the geometry from Fig. 2. However, there are some north–south baselines, such as the one between $(lat,\, \delta lon) = (20,\, 0)$ and $(80,\, 0)$, where the average performance based on the geometry investigation yields a potential mean formal error of ${3.1}\,{\mu } \hbox {s}$, while the impact of erroneous polar motion a priori information is also below 80 microseconds per milliarcsecond-error in polar motion a priori information, i.e., below ${4}\,{\mu } \hbox {s}$ for an error of ${50}\,{\mu } \hbox {as}$ in polar motion.

A more detailed discussion on the impact of polar motion a priori values, as well as other a priori information, is planned to be part of a future study. Here we would like to keep the main focus on the geometry aspect of the baselines.

## Theoretical considerations

### Partial derivatives

In the case of VLBI *Intensives* with typically only two stations observing for one hour, the number of observations, which can be performed, is relatively small (e.g., 20 to 40 for legacy SX stations, 50-60 for VGOS stations). Thus, only the most important parameters can be estimated during the analysis to still receive a reasonably high redundancy. Usually, the estimated parameters are one clock offset (*CL*0) and clock rate (*CL*1) for the remote station only, one zenith wet delay ($ZWD_{a,b}$) offset per station, and, obviously, a dUT1 parameter. Consequently, in a two-station *“Intensive”* session, there is a total of five unknown parameters to be estimated. The corresponding partial derivatives are listed in Eqs. 11–14.

11 $$\begin{aligned} \frac{\partial \tau }{\partial CL0}&= 1 \end{aligned}$$

12 $$\begin{aligned} \frac{\partial \tau }{\partial CL1}&= t - t_0 \end{aligned}$$

13 $$\begin{aligned} \frac{\partial \tau }{\partial ZWD_{a,b}}&\approx \frac{1}{\sin \varepsilon _{a,b}} \end{aligned}$$

14 $$\begin{aligned} \frac{\partial \tau }{\partial UT1}&\approx - \frac{1}{c} \cdot 1.00273 \cdot \cos \delta \nonumber \\&\quad \cdot \left( (x_b - x_a) \cdot \sin h_G + (y_b - y_a) \cdot \cos h_G\right) \end{aligned}$$

Equation 13 is only an approximation since in reality, a proper mapping function, such as the Vienna Mapping Function (Böhm et al. 2006), is used to convert the zenith wet delay to arbitrary elevation angles $\varepsilon$ instead of $\frac{1}{\sin {\varepsilon }}$. However, for the discussion in this work, it is sufficient to use the approximation.

A similar generalization holds for Eq. 14. For easier interpretation within the discussion of this work, a simple form using the old nomenclature is used to express the partial derivative of $\tau$ with respect to dUT1 instead of the new formulation based on the Earth rotation angle of the post 2000.0 paradigm (Petit and Luzum 2010, and references therein). Here, *c* is the speed of light and $\delta$ is the declination of the radio source while $x_a$, $x_b$, $y_a$ and $y_b$ are the coordinates of the two telescopes *a* and *b* in the equatorial plane. The z components of the telescopes’ coordinates do not appear and thus do not play any role. The Greenwich Hour Angle $h_G$ (Mueller 1969) is defined as the difference between the Greenwich Apparent Sidereal Time (*GAST*) and the Right Ascension of the observed radio source ($\alpha$).

15 $$\begin{aligned} h_G = GAST - \alpha \end{aligned}$$

### Dependency of $\partial \tau / \partial dUT1$ on source position

To understand why certain baselines do not work as expected and why a judgment solely based on east–west orientation does not reflect the reality properly, it is necessary to further investigate the partial derivatives of $\tau$ with respect to dUT1. In a least-squares adjustment, in order to distinguish estimated parameters from each other it is critical that their partial derivatives are different and variable to avoid correlations between these parameters. As an analogy, one can think about the problem in distinguishing tropospheric zenith wet delays, station heights, and clock offsets (Nothnagel et al. 2002; Schuh and Böhm 2013). When comparing the partial derivatives of the unknown parameters, in particular Eqs. 11 and 14, it is necessary to derive different dUT1 partial derivatives to distinguish them from the constant clock partial derivatives.

When investigating Eq. 14, one can see that a variation of $\frac{\partial \tau }{\partial dUT1}$ on a baseline is only achievable through varying source declination $\delta$ and, in particular, its right ascension $\alpha$ (expressed through $h_G$, see Eq. 15). Observing sources with different $\alpha$ is more important than different $\delta$, because $\delta \in [-90,90] \rightarrow \cos {\delta } \in [0,1]$ while $\alpha \in [0,2\pi ) \rightarrow \sin {h_g} \hat{=} \sin {\alpha } \in [-1,1]$. Additionally, one can conclude that sources near the celestial poles result in partial derivatives of $\tau$ with respect to dUT1 close to zero because of the term $\cos {\delta }$ and observations of sources near the celestial equator provide the highest partial derivative values. Furthermore, it is evident that a large extension of the baseline on the xy-plane is beneficial to receive large $\frac{\partial \tau }{\partial dUT1}$ values in an absolute sense.

**Fig. 4:**
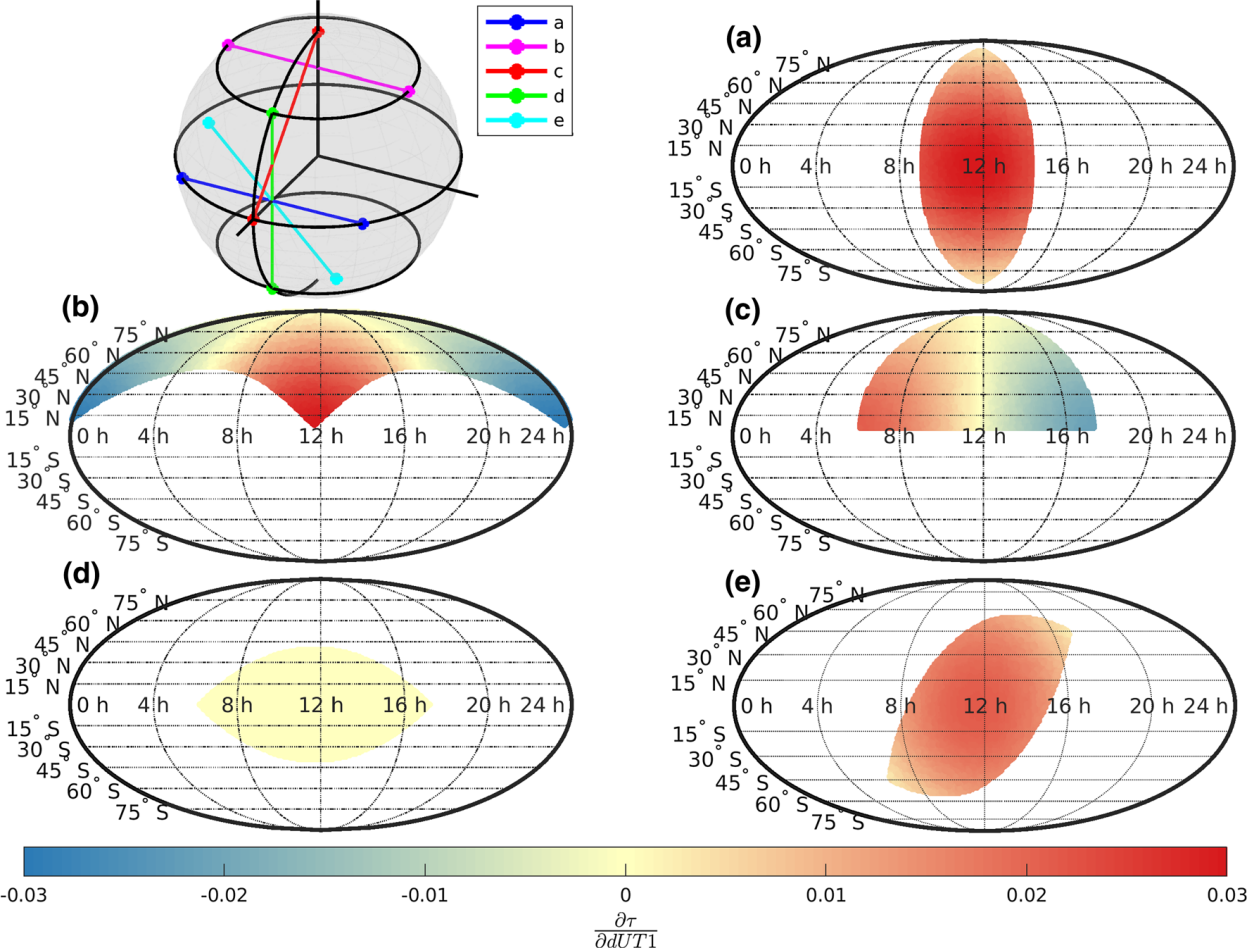
Partial derivative of $\tau$ with respect to dUT1 as a function of baseline orientation and radio source right ascension $\alpha$ and declination $\delta$

Figure 4 visualizes $\frac{\partial \tau }{\partial dUT1}$ of five equally long baselines ($\approx {9000}\hbox { km}$) with different orientations and a cut-off elevation of $5^\circ$ as a function of $\alpha$ and $\delta$.

It is to note that the area of commonly visible sky is of identical size for all baselines. Visually it might look like that the commonly visible sky is smaller in some cases but this is only related to the pseudo-cylindrical Mollweide projection that was used.

Figure 4a depicts the result for the poorly performing equatorial east–west baseline, even though it is ${9000}\hbox { km}$ long. The station coordinates are $$\begin{pmatrix} lat,\, lon \end{pmatrix} = \begin{pmatrix} 0,\, -45 \end{pmatrix}$$ and $$\begin{pmatrix} 0,\, 45 \end{pmatrix}$$. It can clearly be seen that only sources at a relatively narrow $\alpha$-band are commonly visible by both stations. This results in a very small variety of the partial derivatives of $\tau$ with respect to dUT1 that could potentially be observed, which explains why this type of baseline does lead to poor dUT1 precision in Fig. 2.

Figure 4b depicts $\frac{\partial \tau }{\partial dUT1}$ of the same baseline, rotated around the y-axis by 90 degrees. Thus, the station coordinates are $$\begin{pmatrix} 45,\, -90 \end{pmatrix}$$ and $$\begin{pmatrix} 45,\, 90 \end{pmatrix}$$. Although the orientation of the baseline in space is identical to case 4a, the commonly visible sky now spans the whole northern part of the celestial frame. Therefore, sources with different $\alpha$ are visible. This leads to a high variety in the partial derivatives and thus to a good result as highlighted in Fig. 2. Additionally, it is to note that the minima and maxima of $\frac{\partial \tau }{\partial dUT1}$ are located exactly at the corners of the commonly visible sky. Considering that high variability of partial derivatives is beneficial to achieve high precision, this explains the findings of Uunila et al. (2012) and Baver and Gipson (2015).

Figure 4c visualizes the partial derivatives of a north–south baseline with the station coordinates being $$\begin{pmatrix} 90,\, 0 \end{pmatrix}$$ and $$\begin{pmatrix} 0,\, 0 \end{pmatrix}$$ and a length of the equatorial component of ${6370}\hbox { km}$. It can be seen that the variability of the partial derivatives is significantly higher compared to case 4a but also smaller than 4b. The latter is due to the shorter equatorial projection also reflected in the lack of dark red and blue areas. Similar to before, the maximal variation is found by comparing the partial derivatives of sources located at the corners of the commonly visible sky.

Figure 4d shows the partial derivatives of a north–south baseline parallel to the Earth rotation axis located between $$\begin{pmatrix} 45,\, 0 \end{pmatrix}$$ and $$\begin{pmatrix} -45,\, 0 \end{pmatrix}$$. It can be seen that the partial derivatives are constant and close to zero for all right ascensions and declinations. Since in this baseline $x_a = x_b$ and $y_a = y_b$, $\frac{\partial \tau }{\partial dUT1}$ becomes zeros (Eq. 14). Therefore, the baseline is not sensitive to dUT1 and fails to measure it, as can be seen in Fig. 2.

Finally, 4e concludes with pointing out the result of a baseline centered around the equator. For this purpose, baseline 4a was rotated by $-45^\circ$ around the x-axis. The new station coordinates are $$\begin{pmatrix} 30,\, \approx -35 \end{pmatrix}$$ and $$\begin{pmatrix} -30,\, \approx 35 \end{pmatrix}$$. Here, it can be seen that the right ascension span is also quite small, explaining the poor performance of these baselines in Fig. 2.

It is to note that in the case of equatorial baselines (Fig. 4a) or baselines centered around the equator (Fig. 4e) the corners of the commonly visible sky do not provide alternating signs and thus do not span the maximum possible spread of achievable dUT1 partial derivatives. However, it is still advisable to make use of the scheduling approach described in Sect. 2.2 and Appendix A. As it will be further discussed in Sect. 4.3, it is beneficial to observe sources providing a large spread of dUT1 partial derivatives values. For equatorial baselines, this would mean observing sources at the center of the commonly visible sky in conjunction with sources at the corners of the commonly visible sky. Standard scheduling algorithms mostly focus on the center of the commonly visible sky and struggle to add observations to sources located at the corners of the commonly visible sky (Uunila et al. 2012). The newly proposed algorithm tries to better include these corners into the schedule. However, these special observations are only introduced on a fixed time interval (in the case of this study every 15 minutes). In between these special observations, the standard scheduling algorithm is used instead, which focuses on the center of the commonly visible sky, leading to a schedule with observations covering a large spread of dUT1 partial derivatives values.

### Comparison of *Intensive* baseline quality metrics

**Fig. 5:**
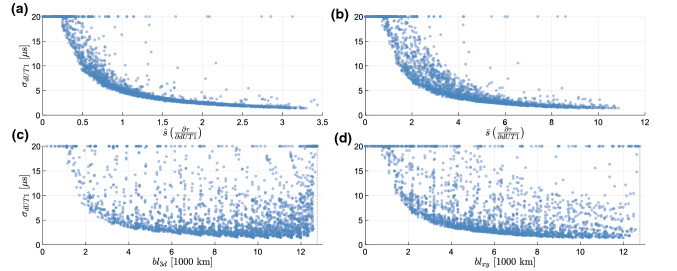
Mean formal error of dUT1 plotted against the effective spread (a equation 16) and total spread (b equation 17) of $\frac{\partial \tau }{\partial dUT1}$ and the baseline length (c equation 18) as well as its projection on the xy-plane (d equation 19). Formal errors of $>{20}\,{\mu } \hbox {s}$ are displayed at ${20}\,{\mu } \hbox {s}$

In an attempt to characterize the importance of certain key dependencies, we also looked at the distribution of all results of Sect. 3 with respect to baseline length, equatorial projection of the baseline as well as the variability of $\frac{\partial \tau }{\partial dUT1}$ in terms of total spread and effective spread. In Fig. 5, $\sigma _{dUT1}$ are plotted against these four metrics.

Since baselines capable of determining dUT1 with high precision are more interesting than the ones providing poor precision, only a $\sigma _{dUT1}$ range between 0 and ${20}\,{\mu } \hbox {s}$ is visualized while formal errors of $> {20}\,{\mu } \hbox {s}$ are displayed at ${20}\,{\mu } \hbox {s}$. To investigate the relation between $\sigma _{dUT1}$ and $\frac{\partial \tau }{\partial dUT1}$, the effective spread of the partial derivatives (${\hat{s}}$, Eq. 16) and the total spread (${\tilde{s}}$, Eq. 17) are calculated.

16 $$\begin{aligned} {\hat{s}}(x)&= \sqrt{\frac{\sum _i^n \left( x_i - \bar{x} \right) ^2}{n}} \end{aligned}$$

17 $$\begin{aligned} {\tilde{s}}(x)&= \max (x)-\min (x) \end{aligned}$$

For comparison, the connection with the baseline length is further investigated by assessing the total 3d-baseline length (eq 18) and its projection on the xy-plane (eq 19).

18 $$\begin{aligned} bl_{3d}&= \sqrt{(x_b-x_a)^2 + (y_b-y_a)^2 + (z_b-z_a)^2 } \end{aligned}$$

19 $$\begin{aligned} bl_{xy}&= \sqrt{(x_b-x_a)^2 + (y_b-y_a)^2} \end{aligned}$$

Furthermore, Table 2 lists the correlation coefficients between these metrics and $\sigma _{dUT1}$.

**Table 2:** Correlation between $\sigma _{dUT1}$ and different metrics based on the partial derivatives of $\tau$ with respect to dUT1 and the baseline length. The first part lists the correlations between the values as depict in Figure 5. The second part lists the correlations of the reciprocal values leading to an even higher correlation

	${\hat{s}}\left( \frac{\partial \tau }{\partial dUT1} \right)$	${\tilde{s}}\left( \frac{\partial \tau }{\partial dUT1} \right)$	$bl_{3d}$	$bl_{xy}$
$\sigma _{dUT1}$	−0.7908	−0.7679	−0.2073	−0.4801

	1/${\hat{s}}\left( \frac{\partial \tau }{\partial dUT1} \right)$	1/${\tilde{s}}\left( \frac{\partial \tau }{\partial dUT1} \right)$	1/$bl_{3d}$	1/$bl_{xy}$
$\sigma _{dUT1}$	0.9459	0.8951	0.3803	0.6373

By comparing the dependency between the parameters visualized in Figure 5a and 5b with 5c and 5d, as well as the correlation values provided in Table 2, it is evident that the dependency between the baseline length and $\sigma _{dUT1}$ is way smaller than the dependency between $\sigma _{dUT1}$ and the partial derivatives of $\tau$ with respect to dUT1. Thus, an *“Intensive”* baseline quality assessment should be performed based on the possible spread of $\frac{\partial \tau }{\partial dUT1}$ instead of the baseline length and its east–west orientation. It should be noted that the equatorial components of the baseline and thus its east–west length are automatically included in the partials for any baseline due to Eq. 14.

The effective spread ${\hat{s}}\left( \frac{\partial \tau }{\partial dUT1} \right)$ is the best metric to judge the suitability of baselines for *“Intensive”* sessions. In Fig. 5a, there are some outlier values with a large ${\hat{s}}\left( \frac{\partial \tau }{\partial dUT1} \right)$ but also a large $\sigma _{dUT1}$. These can be explained by the numerical problem of the least-squares adjustment. The right most three values, around ${\hat{s}} = 3.12$, 2.85 and 2.74 with $\sigma _{dUT1} > {20}\,{\mu } \hbox {s}$, correspond to the baselines $$\begin{pmatrix} 0,\, 0 \end{pmatrix}$$ – $$\begin{pmatrix} -20,\, 180 \end{pmatrix}$$, $$\begin{pmatrix} 10,\, 0 \end{pmatrix}$$ – $$\begin{pmatrix} -30,\, 180 \end{pmatrix}$$ and $$\begin{pmatrix} 20,\, 0 \end{pmatrix}$$ – $$\begin{pmatrix} -40,\, 180 \end{pmatrix}$$. These are very long and thus hardly observable baselines. Depending on the source constellation, it might be that there are too few visible sources to produce a suitable schedule with enough observations explaining the poor performance. Figure 5d and especially 5c further highlight that extremely long baselines (beyond ${12\,000}\hbox { km}$) will provide bad dUT1 sensitivity due to the lack of commonly visible sources.

## Conclusions

Within this work, we provided a global evaluation of VLBI baselines for the rapid determination of dUT1 through *“Intensive”* sessions. For this purpose, almost 3000 baselines between stations distributed on a regular $10 \times 10$ degree grid were simulated and investigated. Besides confirming already known facts, e.g., that observations at the corners of the mutually visible sky are most important for the determination of dUT1, new additional insight could be gained.

So far, it was common knowledge that long east–west baselines are suited best for the determination of dUT1. However, we prove that this statement is over-simplistic and not generally valid.

Especially, equatorial baselines or baselines with a center located on the equatorial xy-plane are not suited to provide high-precision dUT1 values. The reason for this was identified as the limitation of commonly visible sources with varying right ascension angles and the resulting lack in the variability of $\frac{\partial \tau }{\partial dUT1}$. Thus, we recommend that *“Intensive”* baselines should not be evaluated based on their length or east–west extension alone but rather on their capability to perform observations with varying partial derivatives of $\tau$ with respect to dUT1.

Furthermore, it is shown that north–south baselines are also capable of determining dUT1 with reasonable accuracy, in case the baseline orientation is significantly different from the Earth rotation axis, for example, if one station location is close to the polar region while the second station is located close to the equator. However, we also highlighted the need for accurate polar motion a priori information for these north–south baselines since errors in the a priori values propagate to the dUT1 estimates. This, as well as the impact of other erroneous a priori information, will be further investigated in a follow-up study.

## Figures and Tables

**Fig. 6:**
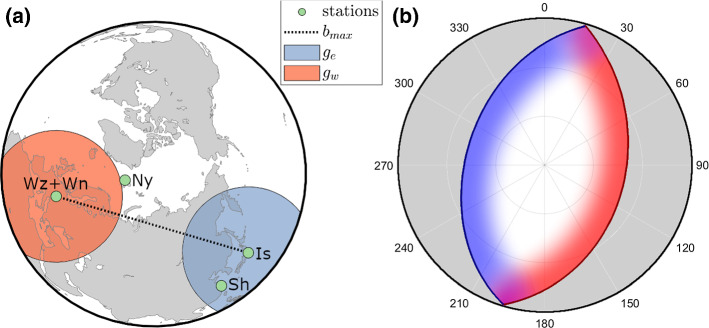
**a** Example of a station grouping for a five station INT3 session. The circles represent the catchment areas of the grouping with a radius of $0.33\cdot b_{max}$. **b** Visible sky as seen from the central point of baseline Wz-Is in a stereographic projection. Gray areas are not visible by both stations simultaneously. Low elevation areas are color-coded—red for Wz, blue for Is. The corners of the commonly visible sky correspond with the areas of low elevation for both stations

## Data Availability

The datasets generated during and/or analyzed during the current study are available from the corresponding author on reasonable request. The software that was used to generate the results is publicly available at https://github.com/TUW-VieVS/VieSchedpp.

## Appendix A

In this publication, we have demonstrated the importance of observations of sources located at the corners (cusps) in the sky which is simultaneously visible by the two telescopes of a standard single-baseline *Intensive* session. For the development of a general *Intensive* scheduling algorithm that makes use of this fact, it is necessary to generalize the problem to a multi-baseline network since some *Intensive* programs, such as INT3, observe with up to five stations. For this reason, it is necessary to look not only at the mutually visible sky of one baseline but to take into account the simultaneously visible sky of the whole network.

To identify the corners of the commonly visible sky of the whole network of an (extended) *Intensive* session, we start with reducing the problem to a single artificial baseline. For this purpose, in a first step, we divide the station network into three groups based on their location with one group representing the “eastern” stations ($g_e$), one comprising the “western” stations ($g_w$) and one representing stations that are not in either of the two groups ($g_0$). For a two-station network the grouping becomes trivial with one station being in group $g_e$ and the other being in group $g_w$.

Next, the longest baseline projected onto the xy-plane (equatorial plane) $b_{max}$ is searched. The two stations *a* and *b* forming this baseline are assigned to the groups $g_e$ and $g_w$. Now, the projected baseline lengths between any of the two stations *a* and *b* and the remaining stations are queried. If a projected baseline length is $< 0.33\cdot b_{max}$, this station is assigned to the corresponding group $g_e$ or $g_w$. Thus, all stations that are reasonably close to a station forming the longest baseline will be referred to the corresponding group. If a station is not reasonably close to any of these stations, it becomes an element of group $g_0$.

Figure 6a visualizes the situation for a five station INT3 session. The longest baseline is between WETTZELL (Wz) and ISHIOKA (Is). The blue circle visualizes the area of $g_e$ including Is. In this example, station SESHAN25 (Sh) would also be assigned to group $g_e$. The red circle depicts the area of $g_w$ including Wz. Here, station WETTZ13N (Wn) would also be part of group $g_w$. Station NYALES20 (Ny) is slightly outside the $g_w$ radius, thus it gets assigned to group $g_0$.

The decision, whether a source is located in the corner of the mutually visible sky, can now easily be made based on the elevation angles in a special way. For this purpose, the mean source elevation angles from the stations in group $g_e$ and $g_w$ are calculated. A source is located at the corner of the mutually visible sky if the mean elevation angles of both groups are reasonably small (e.g., $< 15^{\circ }$). Figure 6b visualizes why this is the case based on a single baseline example between Wz and Is. The graph is a stereographic projection of the visible sky above the baseline midpoint as first devised in Nothnagel and Campbell (1991). By this, it can be depicted conveniently how the visibility is restricted due to the horizon of the two stations. Low elevation areas of the westerly station (Wz) projected onto the baseline mid point are depicted in red color, while the low elevation areas of the easterly station (Is) are visualized in blue color. Gray areas are not visible by both stations simultaneously. It can clearly be seen that the corners of the commonly visible sky are the areas where both stations have small elevation angles—in this example at azimuth $\approx$ 15 degrees and $\approx$ 195 degrees.

Within the implementation of the *Intensive* scheduling algorithm into VieSched++, the likelihood of scheduling the source with the smallest average elevation $\varepsilon _{min}$ is increased by a factor of 1000. The likelihood of scheduling other sources that also have a reasonable small average elevation $\varepsilon$ is also increased by a factor of $1000 \cdot \frac{\varepsilon -\varepsilon _{min}}{\varepsilon _{break}-\varepsilon _{min}}$ where $\varepsilon _{break}$ is selected in a way that approximately the four best sources have an increased likelihood of getting scheduled. Thus, the improved likelihood linearly decreases with increased average elevation. Additionally, it is to note that only sources located at the opposite corner than the one observed before get an increased weight. For the first scan, sources in both corners get an increased likelihood.

This particular approach of increasing the likelihood of scheduling sources is used because it is always beneficial not to force the software into a decision by using strict rules. One obvious reason for this is that the source that is in the best location based on the minimum elevation might be very faint and thus difficult to observe in a short integration time, while there might be another very bright source with only a slightly higher average elevation angle that would be way better to observe. Thus, the scheduling software should have the freedom to decide that it is better to go for the brighter source that is a bit further away from the optimal position instead of the faint source that is in the best position.

As described in Sect. 2.2, increasing the likelihood of scheduling scans of sources located at the corners of the mutually visible sky is only done every $\delta t_c$ seconds, with $\delta t_c$ being defined by the user. In between these scans, the software is following the standard geodetic scan selection procedure as described in Schartner and Böhm (2019). This is necessary because typically there are not enough sources located at the corners of the commonly visible sky to generate a full schedule with only these scans. Additionally, the scans scheduled using the standard geodetic procedure help to de-correlate the impact of tropospheric delays and clock drifts. Furthermore, focusing on sources located at the corners of the commonly visible sky alone would result in a lot of slew time and would not be very robust to source losses.

Additionally, the algorithm tries to alternates between the two corners. Thus, it only increases the likelihood of sources located at the opposite corner that was previously observed. In the case of the first scan, which is always selected based on the proposed algorithm, the software is free to choose a source from any of the two corners.

## Footnotes

1 https://ivscc.gsfc.nasa.gov/sessions/.
